# Effects of cascade reservoir systems on the longitudinal distribution of sediment characteristics: a case study of the Heihe River Basin

**DOI:** 10.1007/s11356-021-15760-y

**Published:** 2021-08-11

**Authors:** Yu Wang, Bao-long Li, Juan-juan Liu, Qi Feng, Wei Liu, Xu Wang, Yu-hua He

**Affiliations:** 1 College of Energy and Power Engineering, Lanzhou University of Technology, Lanzhou, ,730050 China; 2 Key Laboratory of Ecohydrology of Inland River Basin, Northwest Institute of Eco-Environment and Resources, Chinese Academy of Sciences, Lanzhou, ,730000 China

**Keywords:** Heihe River, Sediment, Grain size distribution, Cascade reservoir

## Abstract

Spatial variations in grain size parameters can reflect river sediment transport patterns and depositional dynamics. Therefore, 22 surficial sediment samples taken from the Heihe River and its cascade reservoirs were analyzed to better understand the impact of cascade reservoir construction on sediment transport patterns in inland rivers in China. The results showed that the longitudinal distribution of sediment grain size in the Heihe River was significantly affected by the influence of the cascade reservoirs. The retention rate in the cascade reservoir of the Heihe River reached 79% (193.53 Mt/year), which caused most of the fine sand to accumulate in the reservoir, and the sediment fining degree reached approximately 50%. However, the water discharged from the dam caused serious erosion of the riverbed and coarsening of the sediment, and the coarsening degree was approximately 500%. The backwater zone of the reservoir was influenced by both backwater and released water, and the coarsening degree of sediment was approximately 101%. Sedimentary environmental analysis revealed that the characteristics of the sediment grain size in an upstream tributary of the Heihe River were more influenced by source material than by hydrodynamic conditions, while the grain size characteristics of the mainstream sediments were controlled mainly by hydrodynamic conditions. The characteristics of sediment transport in different reaches of the Heihe River were studied, and the results may provide references for the operation of cascade reservoirs and the sediment control of reservoirs.

## Introduction

River sediments are a significant component of river ecosystems created by soil erosion in river basins (Bravard et al. 2014), and their grain size distribution is affected by multiple factors, including sediment sources and hydrodynamic conditions (Snelder et al. 2011; Zhu et al. 2014; Pan et al. 2015). The grain size characteristics of sediments can intensively reflect the local sedimentary environment, such as its hydrodynamic conditions and changes in water levels, sediment transport, and redistribution processes (Folk 1966; Pejrup 1988). Different sedimentary conditions have specific sediment grain size parameters and their assembly characteristics (Sun et al. 2002; Vandenberghe et al. 2018). Therefore, grain size analysis is extensively applied in environmental studies and used to reveal the origin of sediments (Zanella et al. 2012), identify sedimentary environment types (Halls 1967; Friedman 1979), reflect hydrodynamic conditions during deposition, and infer sediment diffusion, transport, and deposition processes (Ma et al. 2014; Rosenberger et al. 2016).

Cascade reservoirs not only generate electricity to alleviate the energy crisis but also control floods and adjust the uneven spatiotemporal distribution of water resources. Nevertheless, such a large artificial lake can have significant negative effects on a river’s hydrodynamic conditions, including water level fluctuations (Lu and Siew 2006; Wang et al. 2013) and decreased velocity (Klaver et al. 2007; Wei et al. 2008). Subsequent to reservoir impoundment, such changes in hydrodynamic conditions significantly affect the compositions and transport fluxes of sediment in the reservoir area and downstream river (Walling and Fang 2003; Willis and Griggs 2003). A global estimate reveals that greater than 50% of basin-scale sediment flux in regulated basins is potentially trapped in artificial impoundments (Vörösmarty et al. 2003). For example, the sediment discharge into the sea has now decreased to almost zero for the Nile River since the building of the High Aswan Dam in 1964 (Frihy et al. 2003; Stanley and Warne 1998). The largest dam in the Narmada River Basin of India traps 60–80% of sediments (Gupta and Chakrapani 2005). The Yangtze annual sediment flux declined from 510 Mt/year in the pre-dam period (1956–1968) to 120 Mt/year during the post-dam period (2013–2015) after the construction of the Danjiangkou and Three Gorges dams in the basin (Yang et al. 2018a; Yang et al. 2021). Moreover, river sediments are important carriers of chemicals such as nutrients, salt, and pollutants. The nutrients adsorbed onto sediment particles, especially phosphorus, will accumulate with the sedimentation of river sediments (Muller et al. 2007), which may lead to algal blooms in reservoir areas (Yang et al. 2018a, b, Zhou et al. 2013). However, the lower reaches of dammed rivers, which have relatively clear water, often experience bed incision and bank collapse; these effects can further adversely impact riparian infrastructure and riverine ecosystems and cause the drawdown of the alluvial water table (Vörösmarty et al. 2003; Minear and Kondolf 2009; Ran et al. 2013).

Most previous reservoir sedimentation studies have mainly focused on exorheic rivers such as the Yangtze (Yang et al. 2014. Luo et al. 2012, Yang et al. 2018b), Yellow (Walling 2006; Wang et al. 2007), Colorado (Vörösmarty et al. 2003), and Mississippi rivers (Meade and Moody 2010; Xu and Yang 2015). For endorheic rivers originating from the Tibetan Plateau in China where the river gradient is greater, the water depth is shallower, and the flow velocity is fast, which makes the sediment transport characteristics of the river even more complicated under the condition of cascade construction; the influence of high-altitude endorheic river dam construction on the sediment transport process has received much less attention. Due to river segmentation and disruption by cascade dams, depositional environments may be derived from different rivers affected by different hydrodynamic conditions. However, a stable water-sediment relationship is an important prerequisite to maintain the aquatic ecosystem balance in inland river basins (Zhang et al. 2020). As such, accurately analyzing basin-wide river sediment erosion and silting by cascade dam construction is central to studies on reservoir management and water resource utilization.

**Fig. 1:**
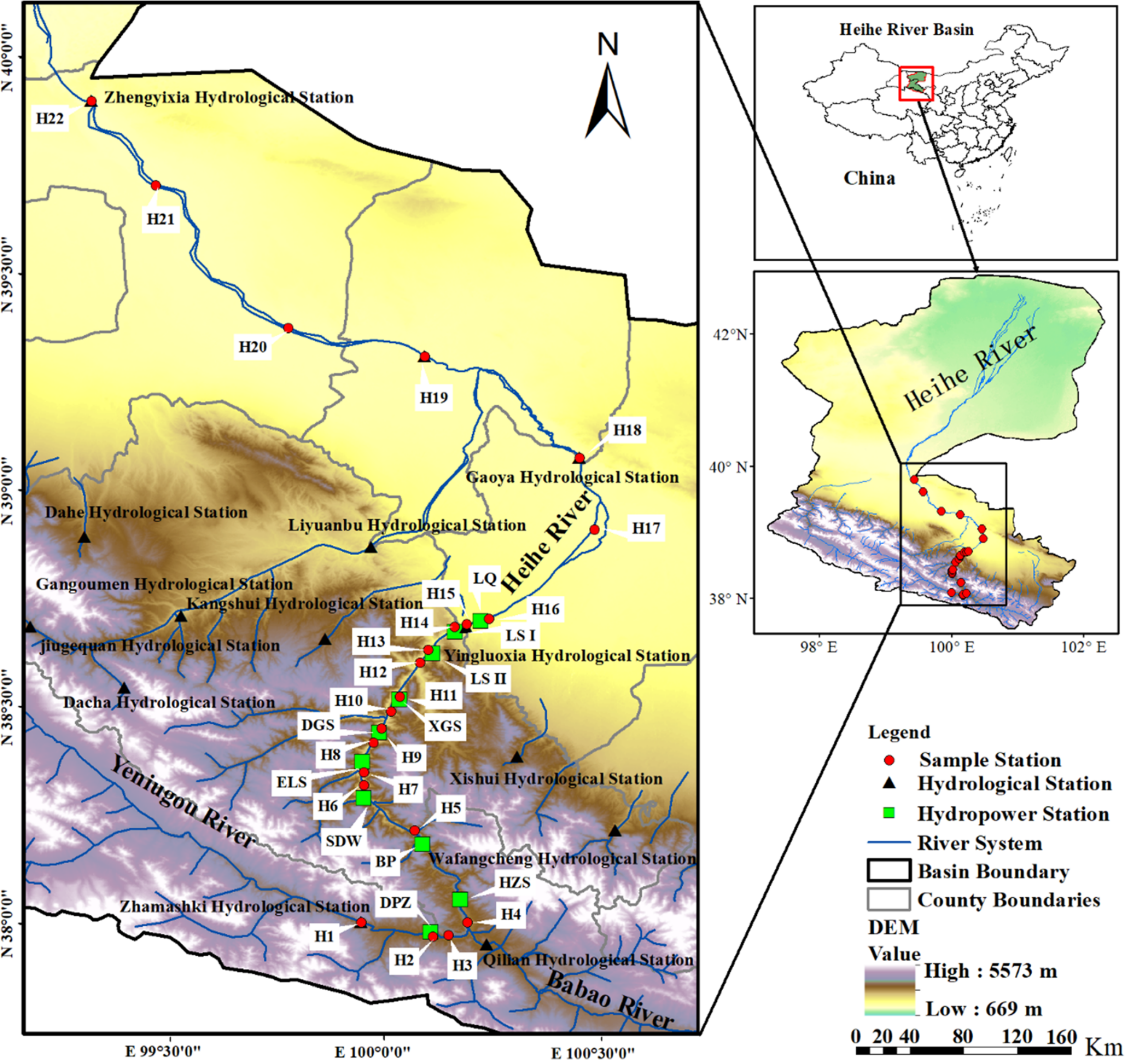
Sketch map of the study area showing the sediment sampling sites (red dots), gauging stations (black triangles), and cascade reservoirs (green rectangle). DPZ, Dipanzi; HZS, Huangzangsi; BP, Baoping; SDW, Sandaowan; ELS, Erlongshan; DGS, Dagushan; XGS, Xiaogushan; LS II, Longshou second; LS I, Longshou first; LQ, Longqu

The Heihe River Basin (HRB), the second largest endorheic river in China, is located in a very important strategic position (Fig. 1). The middle reach of the basin is located on the ancient “Silk Road” and the current Asia-Europe Continental Bridge, and it has become one of the top ten commodity grain bases in China in addition to its long history of agriculture; the Ejina Oasis in the lower reach of the basin is an important ecological security barrier in China (Li et al. 2020). Thus, rivers have undoubtedly played a vital role in the agricultural development of the middle reaches of the HRB and the maintenance of a healthy oasis system in the lower reach. However, a cascade of eight hydropower dams with a river distance of 100 km has already been constructed in the mainstream and tributary reaches of the upper Heihe River, and another larger-capacity reservoir (4.01×10^8^ m^3^) is in the construction stage in the upper reach of the river. The intensive cascade reservoir system in the upstream region of the Heihe River has greatly changed the relationship between water and sediments, and this change has influenced several factors, including the evolution of the river course in the middle and lower reaches, the flow regime from rapid to slow, the riverine ecosystem, and even the development of agriculture in the Hexi Corridor (Wang et al. 2021; Zhang et al. 2010). Previous studies have focused mainly on the analysis of river sediment sources (Derbyshire et al. 1998; Ta et al. 2004; Zhang et al. 2020), and there have been few studies on the influence of human activities on the characteristics of river sediment transport. In this study, we focused on (1) the change in the grain size distribution and compositions of sediments in different reaches, (2) the influence of cascade reservoirs on the grain size distribution and compositions, and (3) the interaction between cascade reservoirs and the deposition environment of the Heihe River. The results of this study provide the spatial variations in basic sediment information for the Heihe Rivers, thereby supporting environmental protection and water resource utilization in this area.

## Study area

The HRB is located in northwestern China, within the range of 96°42′–102°04′ E, 37°45′–42°40′N (Fig. 1). The core drainage area is approximately 143,000 km^2^, with a mainstream length of 821 km. The major tributaries of the upstream Heihe River include the Yeniugou River and Babao River. The two rivers converge at Huangzangsi and flow into the Hexi Corridor. The HRB can be divided into three segments (Zhang and Dong 2015). The upper reach flows within the Qilian Mountains and down to Yingluo Gorge (the outlet of the river in the Qilian Mountains) and is characterized by a steep gradient and a narrow channel. The elevation of the upper reach ranges from 2600 to 4850 m (Table 1), and the mean annual precipitation varies substantially with altitude, increasing from 250 mm in the lower mountainous zone to 700 mm in the higher mountainous zone. The middle reach flows through the Hexi Corridor, from Yingluo Gorge to Zhengyi Gorge. The elevation of this segment decreases from 2600 m to 1350 m, and the mean annual precipitation decreases from 250 mm to <100 mm (Li et al. 2019). The lower reach of the river flows northward from Zhengyi Gorge and terminates in Juyan Lakes (the east and west branches, respectively). The reach has a mean altitude of 1000 m and a mean annual precipitation of <50 mm (Zhu et al. 2008).

**Table 1:** Channel characteristics of the Heihe River

River reaches	River length (km)	Riverbed elevation (km)	River fall (m)	Channel gradient (‰)	Valley width (m)	Flow velocity (m/s)	Riverbed properties
Yeniugou	175	2603–4850	2247	8.5	50–400	3.02	Bedrocks, gravels, and sandy
Babaohe	75	2603–4400	1797	9.3	50–400	3.31	Bedrocks, gravels sandy, and silt
Huangzangsi-Yingluo Gorge	95	1630–2603	900	9.1	20–100	3.23	Bedrocks, gravels sandy, and silt
Yingluo Gorge-Zhengyi Gorge	185	1352–1630	348	2.03	400–800	0.93	Gravels, sandy, silt, and clay

Most of the runoff in the HRB and its tributaries is generated from rainfall and ice/snow melting in the upstream mountainous area; precipitation is the main source (Wang et al. 2009). The hydrological station at Yingluo Gorge (the outlet of the river in the Qilian Mountains) recorded a multiyear average runoff amount (1955–2015) and sediment flux (1968–2015) of 1.65× 10^9^ m^3^/year and 2.06 × 10^6^ t/year, respectively (Wang et al. 2019). The sediment in the Heihe River comes mainly from the upstream mountainous area and is formed by the erosion of surface soils and weathered rocks by precipitation and runoff. Most of the sediments in the middle reaches of the river have been transported there from the upper reaches, but some originate locally when large floods result in riverbed and riverbank erosion. In addition, a small amount of the sediment in the middle reaches is carried in water that returns to the river after being used for irrigation. In general, the properties of the riverbed in the upper reaches of the Heihe River are mainly bedrock, pebbles, and gravel, while they are mainly composed of gravel and sand in the middle and lower reaches (Table 1).

The Heihe River originates on the northeastern margin of the Tibetan Plateau. The upstream mainstream channel has a large elevation drop and contains large amounts of water energy. The upper reaches of the river comprise 106.88×10^4^ kW, with an exploitable capacity of 52.8×10^4^ kW and an annual electricity output of 38.48×10^8^ kW/h. To meet flood control and power generation needs, a series of cascade reservoir and water diversion projects (e.g., Dipanzi, Huangzangsi, Baoping, Sandaowan, Erlongshan, Dagushan, Xiaogushan, Longshou II, Longshou I, and Longqu, Table 2 and Fig. 1) have been conducted in the Heihe River in the past decade. The Longshou I reservoir, built in 2002, was the first reservoir built on the mainstream upper reaches of the Heihe River. The Baopin reservoir, one of the largest reservoirs on the river, began operations in 2012 and had an initial total storage capacity of 2.15×10^8^ m^3^.

**Table 2:** Hydropower development on the mainstream of the upper Heihe River

Hydropower station	Installed capacity (10^4^ KW)	Average annual electricity yield/(×10^8^ KW·h)	Total capacity (10^8^ m^3^)	Operation time
Dipanzi (DPZ)	1.60	0.72	0.0028	September, 2004
Huangzangsi (HZS)	4.90	2.03	4.03	In the construction
Baoping (BP)	12.30	4.10	2.150	July, 2012
Sandaowan (SDW)	11.20	4.00	0.053	May, 2009
Erlongshan (ELS)	5.05	1.74	0.811	September, 2007
Dagushan (DGS)	6.50	2.01	1.410	July, 2009
Xiaogushan (XGS)	10.20	3.91	0.014	July, 2006
LongshouII (LS II)	15.70	5.28	0.862	August, 2004
LongshouI (LS I)	5.20	1.98	0.132	April, 2002
Longqu (LQ)	1.60	0.85	--	December, 2002

## Materials and methods

### Field sampling

After the impoundment of a reservoir, the hydraulic factors of the river such as the water surface slope and flow rate decrease, and the water level in the reservoir area increases. As a river approaches the base level of a reservoir, its water and sediment dynamics are affected by a transitional reach known as the variable backwater zone (Zheng et al. 2019). Sediments in the reservoir and in the variable backwater zone exhibit different erosion and sedimentation characteristics. At low flows, flow deceleration and in-channel sedimentation occur in variable backwater zones, but at high flows, flow acceleration and erosion occur. In addition, sedimentation in the reservoir causes the discharge water flow to erode and scour the downstream riverbed, thereby coarsening the particle size of the river sediments (Dade et al. 2011). To analyze the effects of hydropower development on the erosion and sedimentation characteristics of sediment in the mainstream Heihe River, field sampling in this study was performed at four location types: the natural river, the backwater zone, the lower reaches of the dam, and the reservoir.

Riverbed sediments were collected from 22 sites in the mainstream of the Heihe River (Fig. 1). Nine sites (H_1_, H_3_, H_4_, and H_17_~H_22_) were established on the mainstream of the natural river based on the distribution of hydrological stations, the confluence of rivers, and administrative boundaries. Four sites (H_5_, H_6_, H_15_, and H_16_) were established in the lower reaches of the dam on the mainstream. Four sites (H_7_, H_8_, H_10_, and H_12_) were established in the backwater zone, and five sites (H_2_, H_9_, H_11_, H_13_, and H_14_) were established in the mainstream and tributary of the reservoir. Samples from the natural river, the lower reaches of the dam, and the backwater zone were collected in January 2018. Samples from the reservoir area were collected in August 2018 because the surface of the river was frozen during the January sampling period. The reach of sampling points in the natural river and the lower reaches of the dam are required to be straight, and there is no obvious bend within 100 m of the sampling section. Surface sediments were collected at a distance of 1 m from the waterline of the riverbank. Sediment sampling in the reservoir backwater zone is required to be above the water surface and undisturbed. Sediment samples ranging from 0 to 5 cm on the surface of the riverside beach were collected. In the reservoir, we selected the location of sediment collection near the dam and collected it with the Peterson grab. Three parallel sediment samples were collected from each sampling area using a Petersen mud extractor. The samples were packed into polyethylene bags and taken back to the laboratory for analysis. Moreover, since the reservoir is emptied during the winter dry season to clear out sediments, stratified sampling was performed at three vertical elevations at sampling site H_2_ (labeled H_2-1_, altitude 2691 m; H_2-2,_ altitude 2688 m; H_2-3,_ altitude 2685 m).

### Grain size experiment

The samples were pretreated with 10% H_2_O_2_ and 10% HCl to remove organic matter and biogenic carbonate, respectively. Then, the samples were dispersed with 0.5% (NaPO_3_)_6_ and subsequently dispersed ultrasonically. The grain size was measured with a Malvern Mastersizer 2000 laser particle size analyzer (Malvern Instruments Ltd., UK), with a measuring range from 0.02 to 2000 μm, a particle resolution of 0.01*φ*, and a relative error of <2%.

The grain size and sorting parameters of the sediments were calculated following the formulae devised by Folk and Ward (Folk and Ward 1957). In the calculations, the grain size was measured using phi (*φ*) units (*φ* =  − log_2_*d*, where *d* is the grain size in millimeters) and described using the parameters mean grain size (*M*_*z*_), standard deviation (*σ*), skewness (*S*_*k*_), and kurtosis (*K*_*G*_), as determined by the following formulae:

1 $$ {M}_z=\frac{\varphi 16+\varphi 50+\varphi 84}{3} $$

2 $$ \sigma =\frac{\varphi 84-\varphi 16}{4}+\frac{\varphi 95-\varphi 5}{6.6} $$

3 $$ {S}_k=\frac{\left(\varphi 84+\varphi 16-2\varphi 50\right)}{2\left(\varphi 84-\varphi 16\right)}+\frac{\left(\varphi 95+\varphi 5-2\varphi 50\right)}{2\left(\varphi 95-\varphi 5\right)} $$

4 $$ {K}_G=\frac{\left(\varphi 95-\varphi 5\right)}{2.44\left(\varphi 75-\varphi 25\right)} $$

where *φ*5, *φ*16, *φ*25, *φ*50, *φ*75, *φ*84, and*φ*95 represent the 5th, 16th, 25th, 50th, 75th, 84th, and 95th percentiles, respectively, on the cumulative curve. The grain size parameters were classified by the standards of Folk and Ward (Folk and Ward 1957) (Table 3).

**Table 3:** Grain size grade parameter (Folk and Ward 1957)

Standard deviation	*σ*	Skewness	*S*_K_	Kurtosis	*K*_*G*_
Very well sorted	<0.35	Very negative-skewed	−1.00~−0.30	Very platykurtic	<0.67
Well sorted	0.35~0.50	Negative-skewed	−0.30~−0.10	Platykurtic	0.67~0.90
Moderately sorted	0.50~1.00	Nearly symmetrical	−0.10~0.10	Mesokurtic	0.90~1.11
Poorly sorted	1.00~2.00	Positive-skewed	0.10~0.30	Leptokurtic	1.11~1.50
Very poorly sorted	2.00~4.00	Very positive-skewed	0.30~1.00	Very leptokurtic	1.50~3.00
Extremely poorly sorted	>4.00			Extremely very leptokurtic	>3.00

## Results

### Grain size composition

**Fig. 2:**
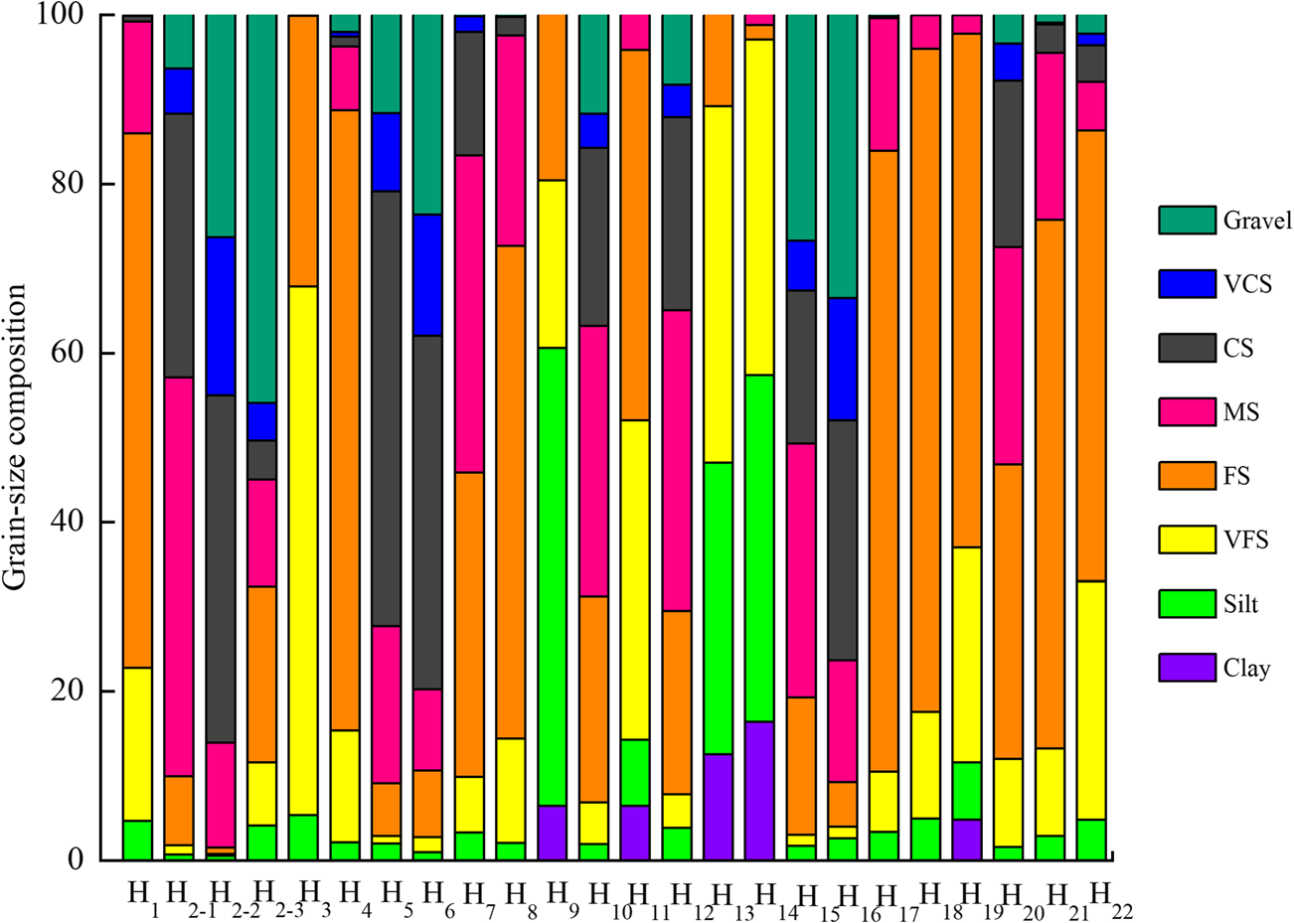
Grain size compositions of river and reservoir sediments at the Heihe River. Clay, >7.64 φ; silt, 3.99–7.64 φ; VFS (very fine sand), 3.00–3.99 φ; FS (fine sand), 2.00–3.00 φ; MS (medium sand), 1.00–2.00 φ; CS (coarse sand), 0–1.00 φ; VCS (very coarse sand), 0–−1.00 φ; gravel, < −1.00 φ

Figure 2 provides the grain size composition of the river (natural river, lower reaches of the dam, and backwater zone) sediments. The mean values from the channel samples show that the natural river sediments were composed mainly of fine sand (59.11%), followed by very fine sand (21.29%) and medium sand (10.44%), with very little very coarse sand or clay content. However, the sediment composition in the backwater zone was relatively balanced between fine sand (35.13%) and medium sand (32.46%), followed by coarse sand (15.46%), very fine sand (6.91%), gravel (5.14%), silt (2.46%), and very coarse sand (2.44%). The sediments from the lower reaches of the dam were composed mainly of coarse sand (34.92%), followed by gravel (23.90%), medium sand (18.17%), and very coarse sand (11.48%), and contained almost no clay. In addition, the sediments from the reservoir of the mainstream were composed mainly of very fine sand (34.86%) and silt (34.18%) and a small amount of fine sand (19.01%) and clay (10.40%), with almost no coarse sand or gravel. The sediment composition in the reservoir of the tributary was much different from that in the reservoir of the mainstream and was composed mainly of coarse sand (26.62%), medium sand (25.08%), and some gravel (24.24%). In general, sediment grain size increased from mostly fine sand in the natural river to mostly coarse sand in the lower reaches of the dam, except within the mainstream reservoirs, which contained mostly very fine sand.

### Grain size parameters of river and reservoir sediments

**Fig. 3:**
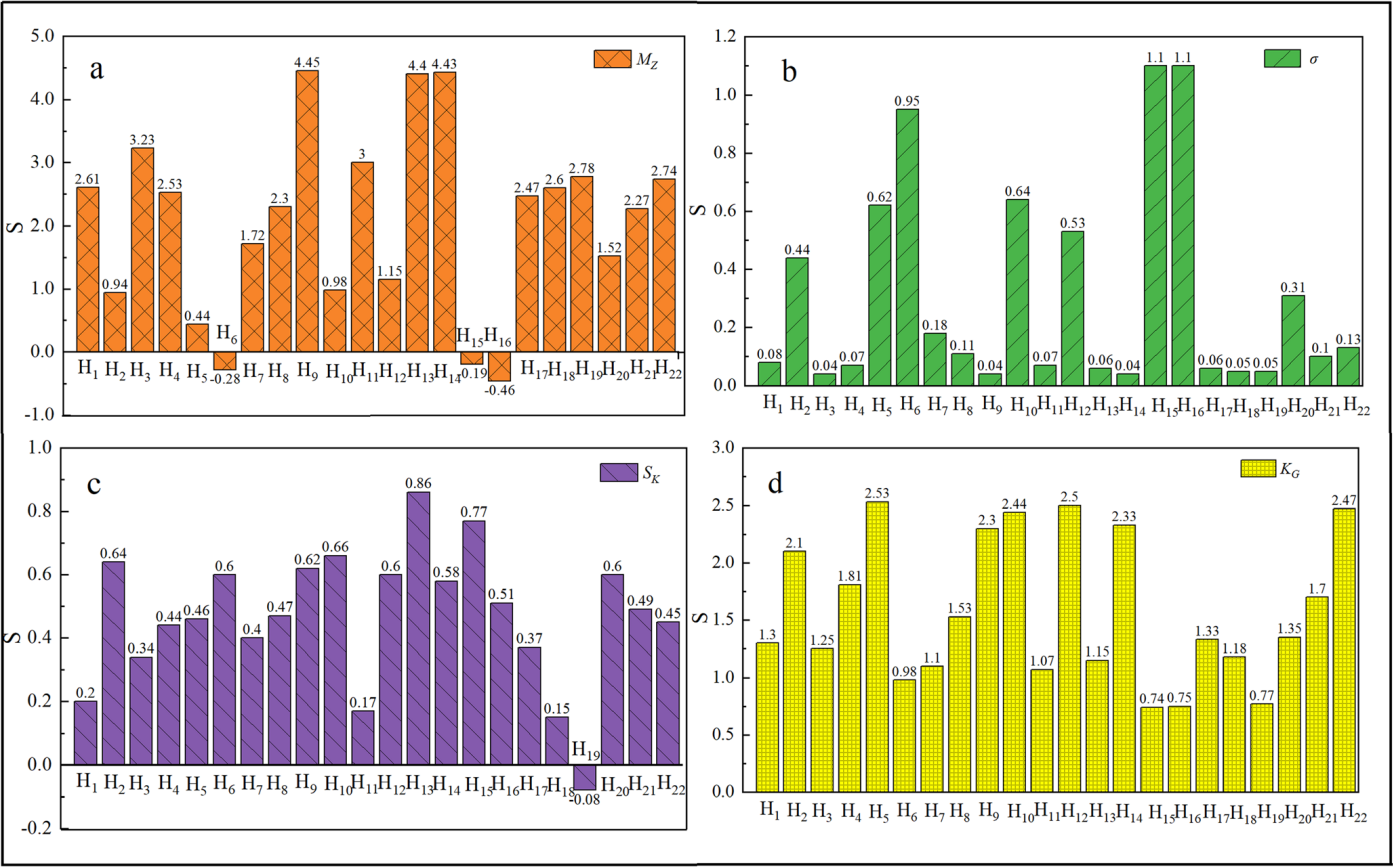
Grain size compositions of river and reservoir sediments at the Heihe River. **a** mean grain size; **b** standard deviation; **c** skewness; **d** kurtosis. H_1_, H_3_, H_4_, and H_17_~H_22_ represent the natural river; H_5_, H_6_, H_15_, and H_16_ represent the lower reaches of the dam; H_7_, H_8_, H_10_, and H_12_ represent the backwater zone; H_2_, H_9_, H_11_, H_13_, and H_14_ represent the reservoir

Figure 3 provides the grain size parameters of the sediments from the different sampling points in the Heihe River. The *M*_*z*_ for the natural river samples averaged 2.52 φ (0.174 mm), with a range of 3.23–1.52 φ (0.107–0.349 mm), indicating that the natural river sediments were composed mainly of fine sand. However, the *M*_*z*_ in the samples from the lower reaches of the dam was −0.06 φ (1.007 mm), with a range of −0.46–0.44 φ (1.376–0.737 mm), which is larger than that in the backwater zone (average of 1.45 φ (0.366 mm), with a range of 2.30–0.98 φ (0.203–0.507 mm)). The sorting values of the natural river sediments measured by *φ* standard deviations (*σ*) ranged from 0.04 to 0.31 (average of 0.10), which is considered to represent very well sorted sediments. The standard deviations of the backwater zone sediments ranged from 0.11 to 0.64 (average of 0.37), and the *σ* value indicated moderately well sorted sediments. However, the standard deviations of the sediments from the lower reaches of the dam ranged from 0.62 to 1.10 (average of 0.94), which is greater than that of the natural river, implying that the sediments at the lower reaches of the dam are less well sorted than those in the natural river. Skewness (*S*_*k*_) is often used to measure the degree of asymmetry of the frequency curve, which is closely related to sorting. A positive skew indicates a relative abundance of coarse grains, whereas a negative skew indicates a relative abundance of fine grains. The *S*_*k*_ of the natural river sediments ranged from −0.08 to 0.60 (average of 0.33) and was mostly positive. The *S*_*k*_ of the backwater zone sediments varied between 0.40 and 0.66 (average of 0.53) and was mostly very positively skewed. The *S*_*k*_ of the lower reaches of the dam sediments varied from 0.46 to 0.77 (average of 0.58), indicating a very positive skew that was more positive than that of the natural river. Kurtosis (*K*_*G*_), or the peakedness, of a grain size distribution compares the degree of sorting of the central population to that of the tails. A negative kurtosis value is described as leptokurtic, in which a large portion exists in the central population, whereas a positive kurtosis value is described as platykurtic, in which a large portion exists in the tails. The mean *K*_*G*_ values of the sediments from the natural river, lower reaches of the dam, and backwater zone sediments were 1.46, 1.25, and 1.89, respectively; these sediment distributions were very leptokurtic largely due to the narrow grain size range.

The *M*_*z*_ of the sediments from the mainstream reservoir averaged 3.92 φ (0.066 mm), with a range of 4.45–3.00 φ (0.046–0.125 mm), which is smaller than that of the sediments from the tributary reservoir (average of −0.13 φ (1.094 mm), with a range of −0.63–0.94 φ (1.548−0.521 mm)). The standard deviations of the sediments from the mainstream reservoir averaged 0.05, which is smaller than the average of those of the sediments from the tributary reservoir (0.87), indicating that the sediments in the mainstream reservoir are very well sorted. The *S*_*k*_ of the mainstream reservoir and tributary reservoir sediments included only two gradations; very positively skewed samples predominated (71.43%), followed by positively skewed samples (28.57%). The *K*_*G*_ values of the mainstream and tributary reservoir sediments were 1.71 and 1.93, respectively, which indicate high leptokurtosis. In addition, the sediment grain size parameters in the tributary reservoir (Dipanzi reservoir) showed strong differences with increasing sediment depth. With increasing sampling depth, *M*_*z*_ increased from 0.94φ (0.521 mm) to −0.63φ (1.548 mm), and the *σ* value changed from 0.44 to 1.26.

**Fig. 4:**
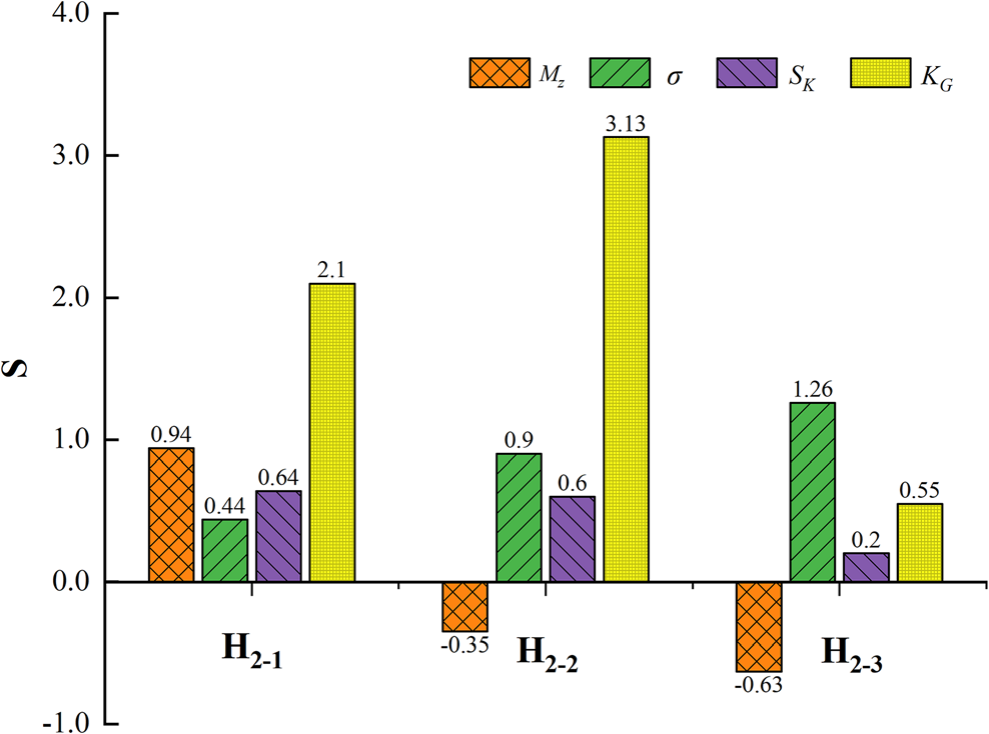
Grain size parameters of different sampling depth in tributary reservoir (Dipanzi reservoir). The H_2-1_ is the surface sediments, the H_2-2_ is the intermediate sediments, and the H_2-3_ is the bottom sediments

*S*_*k*_ became more positive, and *K*_*G*_ gradually flattened (Fig. 4).

### Longitudinal distribution of the sediment grain size parameters in the Heihe Rivers

**Fig. 5:**
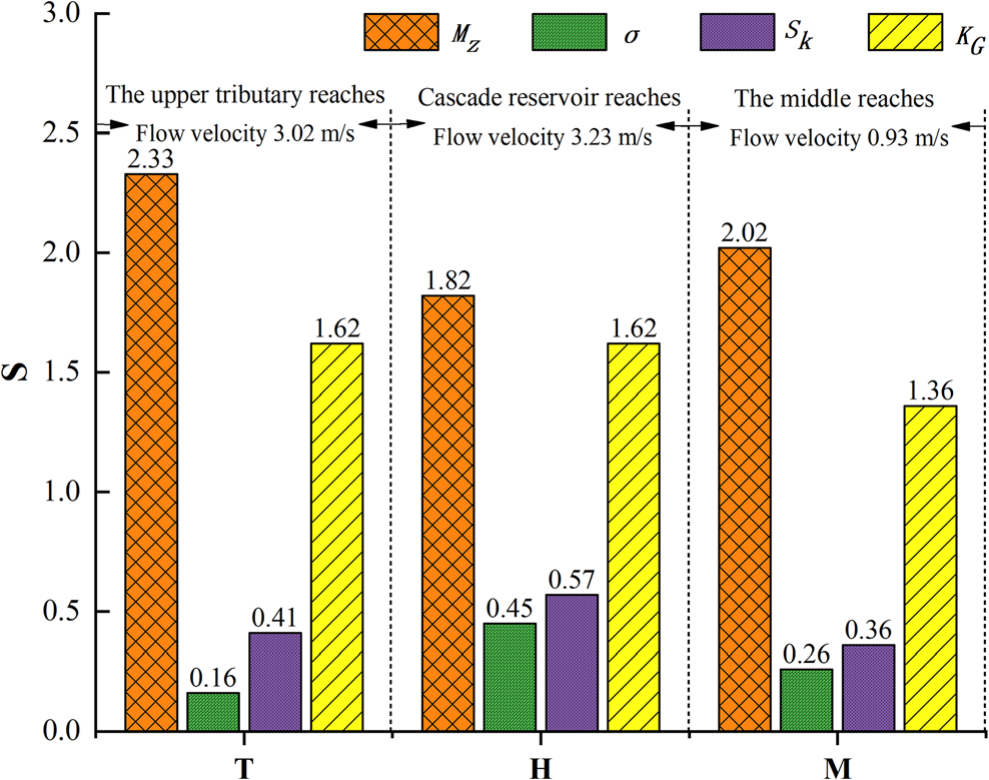
Grain size parameters of Heihe River sediments change along the longitudinal direction. The T is upstream tributary, the H is upper main stream, and the M is middle stream

According to the sampling analysis of the riverbed surface sediment and the cascade reservoir in the Heihe River, the longitudinal distribution characteristics of sediment grain size parameters are shown in Fig. 5. The average *M*_*z*_ values of the Heihe River sediments in terms of longitudinal variability can be ranked as follows: the upper mainstream (cascade reservoir area), 1.82 φ (0.283 mm) > the middle reaches (natural river), 2.02 φ (0.247 mm) > the upper tributary reaches, 2.33 φ (0.199 mm), indicating that the sediment grain size in the upper mainstream is coarser. The sediments in the upper tributary and middle reaches of the Heihe River are well sorted (averages of 0.16 and 0.26, respectively), while the poorest sorting was found in the upper mainstream (average of 0.45). In addition, the *S*_*k*_ of the sediments in the upper tributary reaches and middle reaches was positive (averages of 0.41 and 0.36, respectively), but the *S*_*k*_ of the sediments in the upper mainstream was extremely positive (average of 0.57). The *K*_*G*_ of the sediments showed a leptokurtic trend in the longitudinal direction (averages from the upper tributary reaches to the middle reaches, 1.62, 1.62, and 1.36, respectively). According to the measurement data of sampling position, the Yeniugou River, a tributary of the upstream Heihe River, is characterized by steep gradient and broad-shallow, and the river gradient and average velocity are, respectively, 8.5‰ and 3.02m/s. The river gradient of the cascade reservoir reaches (Huangzangsi-Yingluo Gorge) of Heihe River is approximately equal the former, and the velocity is 3.23m/s (Fig. 5). Theoretically, the *M*_*z*_ of the sediments in two rivers should be the same. However, the sediments in the Yeniugou River had a smaller *M*_*z*_ than those in the cascade reservoir reaches. The longitudinal distribution characteristics of riverbed sediment grain size parameters show that the construction of a cascade reservoir leads to the serious coarsening of sediment and the poorest sorting of the Heihe River.

## Discussion

### Decline and spatial pattern change of the sediment discharge

**Fig. 6:**
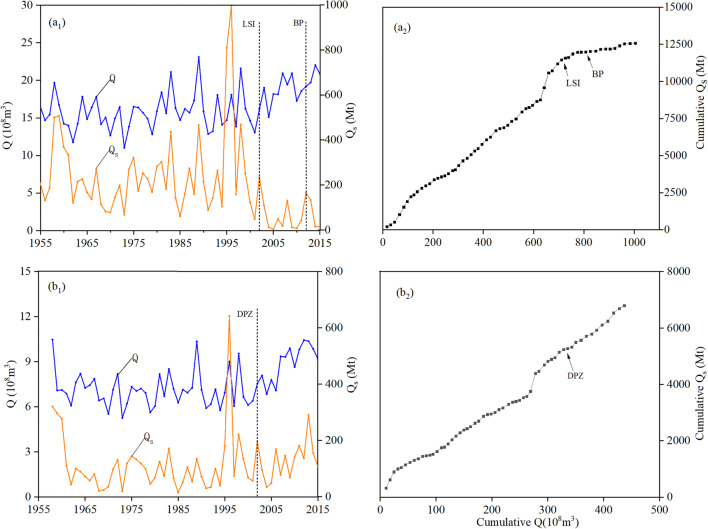
Time series of water (Q) and sediment discharges (Q_S_) and cumulative Q_S_ vs. cumulative Q in the mainstream (**a**) and tributary (**b**)

The sediment in the Heihe River comes mainly from the upstream mountainous area and is formed by the erosion of surface soils and weathered rocks by precipitation and runoff (Zhang et al. 2020). Ice/snow meltwater and precipitation in the high mountain regions are the main water resources of the Heihe River and its tributaries. As the amount and intensity of precipitation increase in the upper reaches of the Heihe River, infiltration flow and surface runoff increase the erosion of the soil, thus increasing the sediment in the river (Wang et al. 2010). According to the observation data of monthly runoff and sediment discharge of two hydrological stations in the Heihe River (Yingluoxia Hydrological Station and Zhimashike Hydrological Station, Fig. 1), the variation trends in the sediment discharge and runoff were similar before 2004 and showed a significant strong correlation between them. However, the Heihe River sediment discharge began to decrease in 2004 when the LS II reservoir and DPZ reservoir in the mainstream and tributary began operation, respectively (Fig. 6). From the pre-dam period (1955–2004) to the post-dam period (2004–2015), the annual mean sediment discharge decreased by 79% in the mainstream and 19% in the tributaries of the Heihe River. According to the sediment transport calculations for the mainstream Heihe River, the cascade reservoirs in the reach from the Huangzangsi to Yingluoxia valleys accumulated 193.53 Mt/year (approximately 79%) sediments since 2004.

### Impacts of cascade reservoir construction on the longitudinal distribution of sediment grain size

**Fig. 7:**
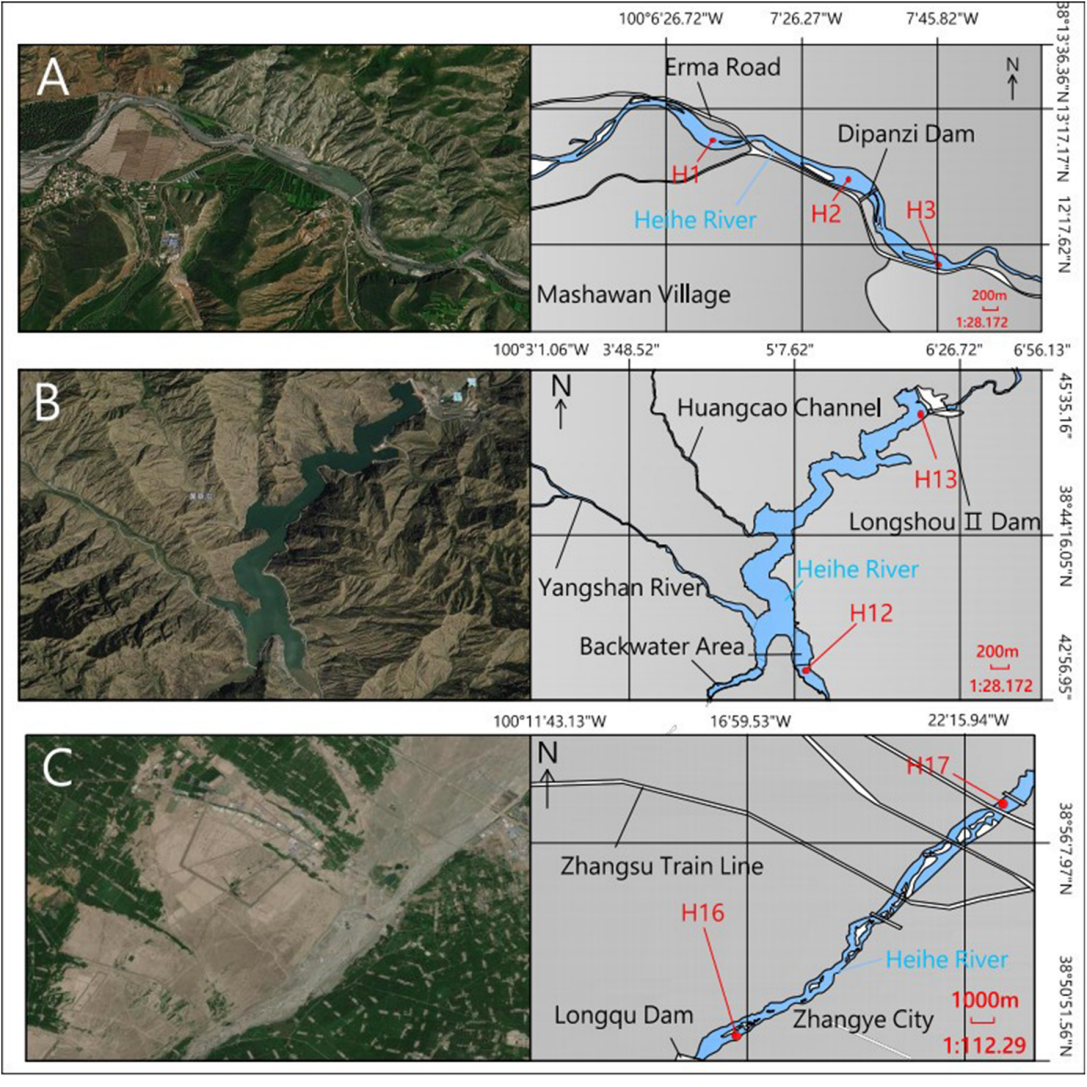
Schematic diagram and velocity of river in different spatial sampling. **A** The sampling position in the upstream tributary (Yeniugou River) and the velocity in H_1_, H_2_, and H_3_ is 3.02 m/s, 0.12 m/s, and 3.03 m/s, respectively; **B** the sampling position in the upper mainstream (cascade reservoir reaches) and the velocity in H_12_ and H_13_ is 0.62 m/s and 0.10 m/s; **C** the sampling position in the middle stream and the velocity in H_16_ and H_17_ is 0.93 m/s and 4.15 m/s, respectively

Rivers continuously transport substances downstream, and these transport processes are essential to driving biogeochemical processes in river ecosystems (Beaulieu et al. 2014; Borges et al. 2018). Dams convert rivers into lentic reservoirs, causing subsequent changes in river hydrodynamic conditions, such as decreased flow velocity and increased hydraulic residence time and suspended particle settlement (Gomez et al. 2001; Maeck et al. 2013). The Heihe River has strong hydrodynamic conditions and sediment transport capacity (Zhu et al. 2008), which result in the sand fraction primarily being sourced from the upstream areas. The upper mainstream (cascade reservoir area) of the Heihe River flows through the alpine valleys where the river gradient is greater (9.1‰), the water depth is deeper, and the average velocity is 3.23 m/s. However, due to river segmentation and disruption by cascade dams, the velocity of the whole reach has changed significantly at different sites. According to the measurement during sampling, the average velocity in the backwater zone and the reservoir decreased to 0.62 m/s and 0.12 m/s, respectively. However, the average velocity of the lower reaches of the dam increased to 4.15 m/s (Fig. 7).

The grain size composition and parameters of sediments are closely related to transport distance, hydrodynamic conditions, and sedimentary environment (Wang et al. 1999). The variation in sediment grain size parameters in different reaches of the Heihe River is caused by the change in local velocity. Compared with the natural river, the mean grain size (*M*_*z*_) of the sediments from the mainstream reservoir decreased from 0.182 to 0.066 mm (the fining degree was approximately 50%), and the sorting (*σ*) decreased from 0.1 to 0.05 (Table 4), which shows that most of the fine sediment was deposited in the mainstream reservoir, and mild flowing conditions created bedding at different sediment depths. The *M*_*z*_ of the lower reaches of the dam sediments was larger than that of sediments in natural rivers, increasing from 0.182 to 1.117 mm, which indicates that the water discharged from the dam has caused serious erosion of the riverbed and coarsening of the sediment, and the coarsening degree was approximately 500%. This effect was also indicated by the sorting values. The standard deviations of the sediment sorting values in the lower reaches of the dam at sampling sites H_15_ and H_16_ and at site H_6_ are as high as 1.10 and 0.95, respectively. Compared with the natural river, the sorting values of sediment grain sizes decreased, with the skewness tending to be very positively skewed and the kurtosis flatter.

**Table 4:** Grain size parameters (average and range) of river and reservoir sediments at the Heihe River (in phi units)

Sampling types	*M*_z_	Change (%)	σ	Change (%)	*S*_K_	*K*_G_
Natural river	2.46	0	0.10	0	0.33	1.46
Backwater zone	1.45	101	0.37	270	0.53	1.89
Lower reaches of dam	−0.16	510	0.94	840	0.58	1.25
Reservoir of tributary	0.94	500	0.87	770	0.56	1.93
Reservoir of mainstream	3.92	−64	0.05	−50	0.48	1.71

^a^Negative value represents a decrease

In addition, the survey in this study showed that the *M*_*z*_ of the backwater zone sediments (0.366 mm) was larger than that of sediments in natural rivers (0.182 mm) but smaller than that of sediments in the lower reaches of the dam (1.117 mm), and the coarsening degree of sediment was approximately 101%. *S*_*k*_ and *K*_*G*_ were also characterized by the same pattern, indicating that the backwater zone of the Heihe River is influenced by both the backwater of the reservoir and the clear water released from the reservoir; both erosion and sedimentation occur. These two influences are the main reason that the grain size parameters of sediments in the backwater zone of the reservoir are between those of natural rivers and those in the lower reaches of the dams.

### Analysis of the sedimentary environment in the Heihe River

Previous studies have shown that changes in material sources and hydrodynamic properties can lead to different depositional environments (Morris and Williams 1999). Conversely, different sedimentary environments can be inferred based on the sediment composition and grain size parameters (Frings 2008; Curtis et al. 2010; Guo et al. 2020). According to Church’s research showing that riverbed sediments tend to fine downstream, the grain size of the riverbed sediments is compatible with the hydrodynamic conditions of the river. The stronger the hydrodynamic conditions are in a river, the larger the *M*_*z*_ of the sediment will be, and vice versa (Church and Kellerhals 1978; Rice and Church 1998). Generally, the more downstream a river is, the lower the slope of the water surface, the lower the flow velocity, and the finer the corresponding riverbed sediments. However, the longitudinal distribution of sediment grain size parameters in the Heihe River determined in this study showed that downstream-fining trends in the sediments were only a macroscopic phenomenon. We found that the longitudinal distribution of the sediment grain size was significantly influenced by sediment material sources and cascade reservoir interception, which makes the trend of sediments toward the downstream fining even more complicated. The total length of the upstream tributary of the Heihe River investigated in this study is approximately 175 km, with a slope of 8.5‰ and a mean flow velocity of 3.02 m/s. The overall length of the middle reach of the Heihe River is approximately 185 km, with a slope of 2.03‰ and a mean flow velocity of 0.93 m/s. The hydrodynamic conditions of the upstream tributary are more intense than those of the middle reaches of the Heihe River, but the *M*_*z*_ of the sediments in the upstream tributaries is smaller than that in the middle reaches. These trends indicate that the grain size of sediments in the upstream tributaries is more significantly affected by material sources than by hydrodynamic conditions. In the upper reaches of the mainstream, the *M*_*z*_ of the sediments in the reservoir was smaller than that in the backwater zone, which means that when the sediment material source was the same, the hydrodynamic conditions in the reservoir of the Heihe River were weaker than those in the backwater zone. Our analysis of sediment grain size parameters in the natural river and the lower reaches of the dam shows that the hydrodynamic conditions in the lower reaches of the dam are stronger than those of the natural river when the sediment source for the river is not substantially different.

## Conclusions

(1) The cascade reservoirs in the upper reaches of the Heihe River strongly impact the longitudinal distribution of sediment grain size. The retention rate in the cascade reservoir of the Heihe River reached 79% (193.53 Mt/year), which caused most of the fine sand to accumulate in the reservoir, and the sediment fining degree reached approximately 50%. However, the water discharged from the dam caused serious erosion of the riverbed and coarsening of the sediment, and the coarsening degree was approximately 500%. In addition, the backwater zone of the Heihe River is influenced by both the backwater of the reservoir and the clear water released from the reservoir. Both erosion and sedimentation occur, and the coarsening degree of sediment is approximately 101%.

(2) Sedimentary environmental analysis showed that the grain size of the sediments in the upstream tributaries of the Heihe River was more significantly affected by material sources than by hydrodynamic conditions; in the mainstream Heihe River, the grain size of the sediments was affected mainly by hydrodynamic conditions.

## Data Availability

The data and materials used in the study are available from the corresponding author by request.
